# Cryo-EM structure of the naked mole-rat ribosome reveals a stabilized split 28S rRNA

**DOI:** 10.1038/s41467-026-75143-0

**Published:** 2026-07-03

**Authors:** Mehmet Gül, Alice Rossi, Christian M. T. Spahn, Gary R. Lewin, Mikhail Kudryashev

**Affiliations:** 1 In situ Structural Biology, Max Delbrück Center for Molecular Medicine in the Helmholtz Association (MDC), Berlin, Germany; 2 Institute for Chemistry and Biochemistry, Freie Universität Berlin, Berlin, Germany; 3 Molecular Physiology of Somatic Sensation Laboratory, Max Delbrück Center for Molecular Medicine in the Helmholtz Association (MDC), Berlin, Germany; 4 Institute of Medical Physics and Biophysics, Charité—Universitätsmedizin Berlin, Corporate Member of Freie Universität Berlin and Humboldt Universität zu Berlin, Berlin, Germany; 5 Charité—Universitätsmedizin Berlin, Corporate Member of Freie Universität Berlin and Humboldt Universität zu Berlin, Berlin, Germany; 6 German Center for Mental Health (DZPG), Partner Site Berlin, Berlin, Germany

**Keywords:** Cryoelectron microscopy, Ribosome

## Abstract

The naked mole-rat *(Heterocephalus glaber)* is a long-lived mammal with resistance to cancer and hypoxia, suggesting the evolution of robust proteostasis networks. The ribosome, central for protein synthesis, is key to cellular stress responses and has an unusual feature: the 28S rRNA split; however, the details of its organization remain unknown. Here, we present high-resolution cryo-EM structures of the naked mole-rat 80S ribosome in four states of the elongation cycle. The structures reveal a conserved overall architecture and rRNA modification landscape compared to other mammals, and provide an atomic-level view of the distinct break in the 28S rRNA. This cleavage event, located in the D6 expansion segment, is structurally stabilized by a network of interactions with surrounding ribosomal proteins, maintaining the integrity of the large subunit. Our comparative analysis revealed that this compensatory network preserves a canonical architecture that is nearly indistinguishable from intact mouse and human ribosomes. These findings resolve the structural basis of this distinct cleavage, showing that it is a stable, integrated feature whose function is likely linked to more subtle regulatory mechanisms, rather than inducing major structural rearrangements.

## Abstract (web summary)

The long-living rodent naked mole-rat has a distinct break in its 28S ribosomal RNA. Here, the authors present cryo-EM structures showing this cleavage is stabilized by surrounding ribosomal proteins, preserving a canonical ribosome architecture.

## Introduction

The naked mole-rat (NMR) is a subterranean rodent with slower than typical mammalian aging, exhibiting extraordinary longevity, sustained fertility, and remarkable resistance to age-related diseases, including cancer and neurodegeneration^1,2^. This distinct biology is coupled with tolerance to extreme environments, particularly severe hypoxia, a condition that would be lethal to most other mammals^3,4^. These physiological traits suggest the existence of highly robust cellular maintenance programs, particularly the network of pathways that maintain protein homeostasis, or proteostasis^5^. The ribosome, the universally conserved ribonucleoprotein complex responsible for protein synthesis, is central to proteostasis. It is a major consumer of cellular energy and a primary control point for managing cellular resources, especially during periods of environmental stress, such as hypoxia, where rapid translational repression is a critical survival strategy. Ribosomes from many species have been extensively studied by structural methods^6–8^, including the detailed understanding of their regulation^9,10^; however, structural insights into the ribosomes of NMRs are missing.

Previous studies described distinct characteristics of the NMR translation machinery. Fibroblasts from NMRs exhibit increased translational fidelity compared to those from mice, and a distinct processing event was identified in the 28S ribosomal RNA (rRNA)^5^. This event, termed a “hidden break,” results from the excision of a ~ 260-nucleotide fragment from the D6 expansion segment during rRNA maturation, leaving two fragments that remain associated within the large ribosomal subunit. This raises a question: how is the structural and functional integrity of the large ribosomal subunit maintained despite a break in its rRNA backbone? The structural basis and functional consequences of this unusual rRNA architecture have remained unknown.

Comparative studies support the view that ribosomal heterogeneity can have functional consequences. For example, in *Plasmodium* species, the presence of distinct stage-specific rRNA alleles with divergent expansion segments enables specialized translation programs during the parasite life cycle^11,12^. While mammalian rDNA is generally homogenized across tandem repeats, subtle sequence and structural variations do exist and may affect translation efficiency and regulation^12^. A high-resolution comparison of naked mole-rat ribosomes with those of related mammals is therefore essential for distinguishing universally conserved features from potential adaptations.

In this work, we used single-particle cryo-EM to determine the structure of the NMR ribosome across multiple functional conformations, enabling a rigorous, quantitative comparison with other mammalian ribosomes^6,13,14^. We determined the structures of the NMR 80S ribosome purified from NMR liver. We provide an atomic-level visualization of the NMR ribosome in four distinct states and describe the break within the 28S rRNA. Our comparative analysis reveals that while the overall ribosome structure is highly conserved compared to other mammals, the cleavage site is a stable, fully integrated feature. A conserved scaffold of ribosomal proteins maintains the rRNA fragments in a canonical conformation, preserving the energetic stability of the region around the split. Our work thus defines the structural basis of a distinct, species-specific feature of the NMR ribosome, demonstrating that the rRNA cleavage is a stable element of the NMR translation machinery, rather than a sign of structural instability.

## Results

### Cryo-EM structures of the naked mole-rat 80S ribosome capture four states of the elongation cycle

**Fig. 1:**
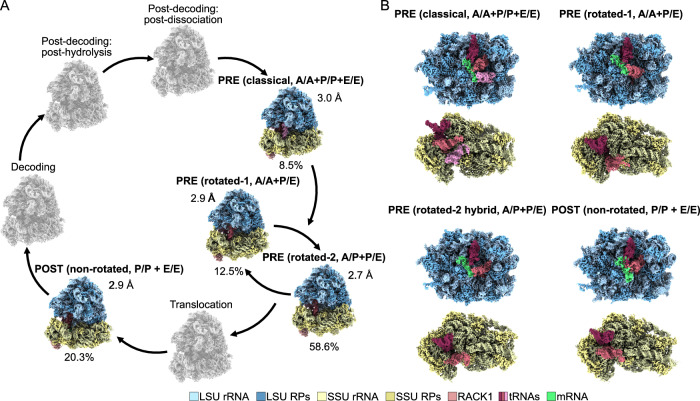
Structural landscape of the naked mole-rat translation elongation cycle. **A** Diagram of the 80S elongation cycle. Colored maps represent states determined by cryo-EM, while gray maps indicate transient states not observed in this study. The percentages and resolutions associated with each map represent the relative particle population and global resolution of each state, respectively. **B** Subunit-level views of the pre-translocation classical (PRE classical), rotated-1 pre-translocation (PRE rotated-1), rotated-2 pre-translocation (PRE rotated-2), and non-rotated post-translocation (POST non-rotated) states. For each state, the 60S (top) and 40S (bottom) subunits are shown with associated mRNA and tRNAs. Color coding for ribosomal components and ligands is shown in the legend below (LSU: 60S, SSU: 40S, RP: ribosomal protein).

To investigate the structural basis of protein synthesis in the NMR, 80S ribosomes were purified from liver tissue and subjected to single-particle cryo-EM. The analysis resulted in four predominant structurally distinct conformational states, representing the intermediates of the translation elongation cycle, a pre-translocation classical state with tRNAs in the A, P and E sites (classical PRE), a rotated-1 pre-translocation state with A/A and P/E tRNAs (rotated-1 PRE), a post-translocation (POST) state with tRNAs in the P and E sites, and a rotated-2 pre-translocation state (rotated-2 PRE) with a rotated SSU and hybrid A/P and P/E tRNAs. The reconstructions had resolutions of 3.0 Å for the classical PRE state, 2.9 Å for the rotated-1 PRE and POST states, and 2.7 Å for the rotated-2 PRE state (Fig. 1, Supplementary Figs. 1, 2, 3, Supplementary Table 1). The quality of the cryo-EM density maps is high throughout the conserved core of both the large 60S (LSU) and small 40S (SSU) subunits, with well-resolved rRNA bases and protein side chains that enabled confident building of atomic models (see Methods).

The four resolved states represent conformationally distinct states of the translation elongation cycle in the naked mole-rat (Fig. 1A). These structures differ primarily by a progressive rigid-body counter-clockwise rotation of the SSU relative to the LSU, often referred to as ribosomal ratcheting, along with tRNA translocation. These conformational changes represent the canonical states of the eukaryotic translation elongation cycle^15–17^.

Consistent with their assignment as distinct functional intermediates, these four structures exhibit different configurations of bound tRNAs that correlate with the SSU rotation (Fig. 1B, Supplementary Fig. 4). In the classical PRE state, tRNAs occupy the classical A/A, P/P, and E/E sites immediately following decoding. In the rotated-1 PRE state, the ribosome initiates the transition to a hybrid state, where the P-site tRNA moves toward the E-site on the 60S subunit while remaining anchored to the P-site on the 40S subunit, resulting in an A/A and P/E configuration. This movement is further resolved in the rotated-2 PRE state, which captures the fully hybrid configuration where both the A-site and P-site tRNAs have translocated their acceptor ends on the 60S subunit, forming A/P and P/E hybrids. Finally, the POST state shows the ribosome returning to a non-rotated configuration with tRNAs occupying the classical P/P and E/E positions.

The capture of these canonical, physiologically relevant conformations confirms the functional integrity of the purified ribosomes. It provides a framework for a detailed comparative analysis of the naked mole-rat translation machinery. Collectively, these observations indicate that the captured PRE and POST states of the naked mole-rat ribosome represent distinct elongation intermediates, differing in the relative orientation of the ribosomal subunits and in the positions of bound tRNAs. The structural features observed here align with conformational changes observed in the translation elongation mechanism^15,16^.

### The naked mole-rat ribosome shares a highly conserved architecture with its mammalian counterparts

**Fig. 2:**
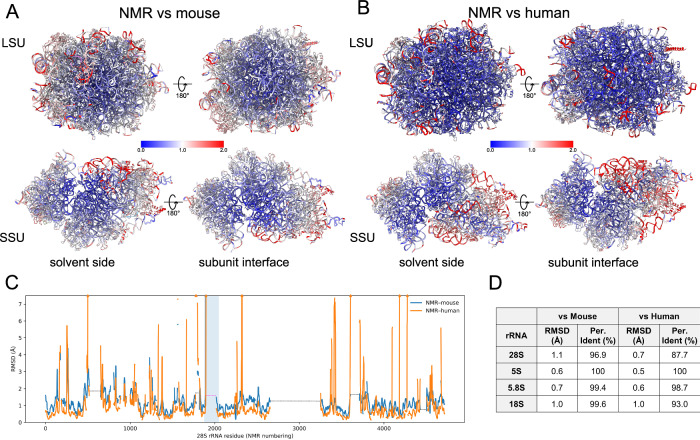
Structural comparison of the naked mole-rat (NMR) and mouse 80S ribosomes in the rotated state. **A** Superposition of the naked mole-rat ribosome and the mouse ribosome subunits, colored by Cα RMSD values. **B** Superposition of the naked mole-rat ribosome and the human ribosome subunits (LSU: 60S, SSu; 40S) colored by Cα RMSD values. **C** RMSD-per-residue plot of the naked mole-rat 28S rRNA with mouse (blue) and human (orange). The unmodelled gaps in the chain are indicated with dashed lines. The 28S rRNA cleavage site is highlighted with a pale blue background. **D** Phosphate backbone RMSD analysis and sequence identity of rRNAs between NMR and mouse/human.

To identify possible species-specific features from the conserved architecture of the mammalian ribosomes, we compared the structure of the PRE rotated-2 state NMR ribosome with the structures from the mouse (*Mus musculus*) kidney (PDB: 7CPU)^18^ and human HeLa cells (PDB: 6QZP)^7^, both resolved to a similar global resolution. To minimize the influence of different structural states, the superpositions were performed for the subunits individually (Fig. 2A, B). In all comparisons, the conserved cores of the LSU and SSU remain uniformly low in deviation, with lower RMSD values, whereas the solvent-exposed loops and flexible loops on the intersubunit face show higher differences. Our analysis using the human structure revealed a high degree of conservation. For the LSU, deviations in the NMR–human comparison are notably low, confirming a near-identical core architecture. Similarly, the SSU shows strong conservation with minimal deviations. Residue-wise analysis of the 28S rRNA (Fig. 2C) now shows that the NMR–human comparison (orange) exhibits a remarkably low baseline deviation, with discrete peaks corresponding primarily to the most flexible, solvent-exposed expansion segments. The comparison with the mouse structure (blue) remains consistent, showing low overall deviation.

The majority of ribosomal proteins exhibit high sequence identity and low Cα RMSD values when compared to both mouse and human orthologs, indicating strong structural homology (Table 1). Deviations are largely confined to the solvent-exposed termini and flexible loop regions of peripheral proteins, consistent with known patterns of interspecies variability^19^ (Fig. 2A). Similarly, analysis of the rRNAs shows that the sequence identities and the core secondary and tertiary structures are nearly identical, with the most significant deviations localized to the tips of several eukaryotic-specific rRNA expansion segments (ESs) (Fig. 2D). This high degree of overall structural conservation underscores the strong evolutionary pressure to maintain the core functional machinery of translation. Within this largely conserved framework, the 28S rRNA region stands out with a distinct structural divergence specific to the NMR.

**Table 1:** Cα RMSD and sequence identity (Per. Ident) comparison of ribosomal proteins between NMR and mouse/human

	vs Mouse		vs Human			vs Mouse		vs Human			vs Mouse		vs Human	
Protein	RMSD (Å)	Per. Ident (%)	RMSD (Å)	Per. Ident (%)	Protein	RMSD (Å)	Per. Ident (%)	RMSD (Å)	Per. Ident (%)	Protein	RMSD (Å)	Per. Ident (%)	RMSD (Å)	Per. Ident (%)
eS24	1.2	98.3	0.6	98.3	eL13	0.6	100	0.6	98.1	uS5	0.5	100	0.5	99.5
uS4	1.0	93.5	0.6	93.5	eS21	0.6	97.6	0.5	92.8	uL23	0.4	100	0.4	100
eS6	0.8	97.6	0.7	97.6	eL34	0.6	100	0.5	99.1	uS7	0.4	94.7	0.5	94.7
eS17	0.8	100	0.7	100	uL29	0.6	100	0.5	98.4	eL32	0.4	100	0.5	100
eS30	0.8	100	0.5	100	uS9	0.5	100	0.5	100	eL20	0.4	100	0.3	99.4
uS10	0.8	99.0	0.6	99.0	uS14	0.5	100	0.5	100	eL31	0.4	100	0.5	100
eS10	0.7	92.7	0.5	92.8	uS2	0.5	100	0.5	100	eL43	0.4	100	0.4	100
eL29	0.7	85.3	0.8	84.0	eL42	0.5	100	0.5	100	uL30	0.4	100	0.4	98.1
eS8	0.7	91.1	0.5	91.1	uL18	0.5	100	0.6	98.3	uL16	0.4	94.4	0.5	89.7
uS3	0.7	100	0.7	99.6	uL15	0.5	100	0.5	97.3	eL15	0.4	100	0.4	100
eS7	0.7	97.3	0.7	98.9	eL8	0.5	94.9	0.6	95.3	eL28	0.4	100	0.5	99.2
eS27	0.7	100	0.7	100	uL3	0.5	100	0.5	98.5	eL41	0.4	100	0.5	100
eL6	0.7	87.1	0.7	84.8	eS1	0.5	100	0.5	100	uS8	0.4	100	0.4	100
eS4	0.7	100	0.5	100	uS13	0.5	100	0.6	100	eL30	0.4	100	0.5	100
eL19	0.6	100	0.4	100	uS11	0.5	100	0.5	100	eL40	0.4	100	0.4	98.0
uL4	0.6	100	0.5	98.0	eS26	0.5	99.0	0.6	99.0	uL14	0.4	100	0.5	100
eS25	0.6	100	0.8	100	eL37	0.5	100	0.5	100	eL18	0.4	100	0.4	96.3
eL21	0.6	100	0.8	99.4	uS15	0.5	100	0.5	100	eL33	0.4	100	0.4	99.0
uS17	0.6	91.8	0.5	91.8	eL14	0.5	100	0.5	92.7	eL39	0.3	100	0.4	100
eL36	0.6	100	0.6	100	uL13	0.5	100	0.4	95.5	eL24	0.3	100	0.4	100
uS19	0.6	100	0.7	100	eL38	0.5	100	0.7	98.6	uL24	0.3	100	0.4	100
eL22	0.6	100	0.8	100	eS19	0.5	100	0.5	99.3	eL27	0.3	100	0.6	100
uS12	0.6	99.3	0.7	99.3	uL5	0.5	100	0.8	100	uL22	0.3	99.3	0.4	98.7
eS28	0.6	100	0.8	100	uL6	0.5	100	0.5	99.0	uL2	0	100	0.4	100

### The 28S rRNA cleavage is a stable feature that does not perturb the local protein environment

**Fig. 3:**
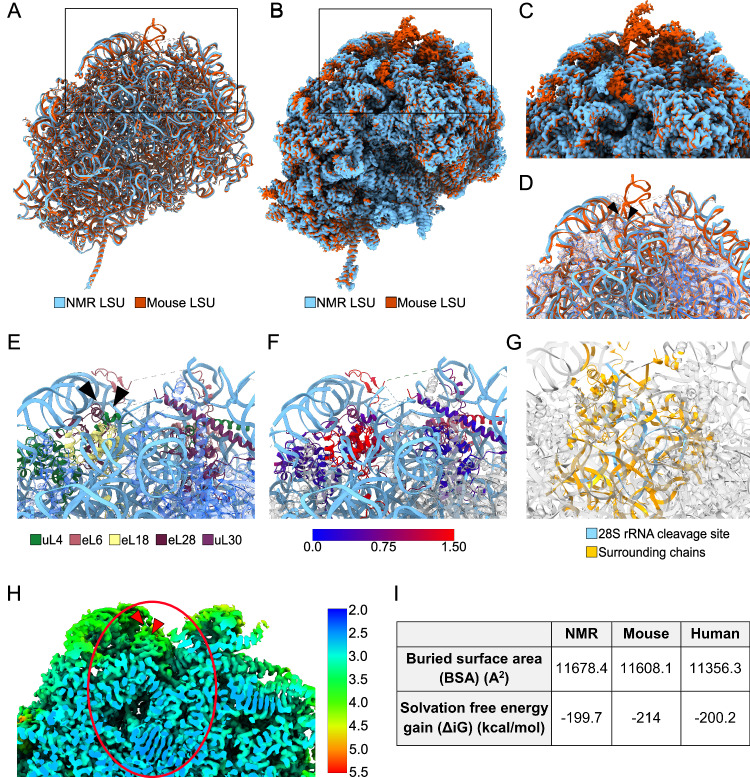
Comparison of structures of the 28S rRNA D6 region in naked mole-rat (NMR) and mouse ribosomes. **A** Overall superposition of the naked mole-rat and mouse large ribosomal subunits (LSU) to visualize the D6 domain of the 28S rRNA. **B** Superposition of the NMR and mouse cryo-EM maps. **C** Close-up view of the D6 cleavage site, with the naked mole-rat map shown in blue and the mouse map in red. The rRNA break points are indicated with arrowheads. **D** Close-up view of the cleavage site on the superposed atomic models of the NMR and mouse ribosomes. **E** Cleavage site in the NMR ribosome, with 28S rRNA shown in light blue and surrounding proteins in different colors. The rest of the proteins are displayed with increased transparency to improve visibility. **F** Cleavage site in the NMR ribosome, with 28S rRNA shown in light blue and surrounding proteins colored by RMSD. A scale bar for RMSD values is provided. **G** The region extracted for PDBePISA analysis is shown in light blue (28S rRNA fragment) and orange (surrounding chains). **H** Close-up view of the D6 cleavage site and its surroundings, colored by local resolution. The rRNA break points are indicated with arrowheads. The region extracted for PDBePISA analysis is annotated with a red ellipse. **I** Buried surface area and solvation free energy gain values for NMR, mouse, and human ribosomes in the NMR 28S rRNA cleavage site.

A key point of comparison is the D6 region of the 28S rRNA, where a 28S rRNA cleavage site has been previously reported in naked mole-rats^5^. Focused examination of the 28S rRNA D6 region revealed a structural discontinuity in the NMR ribosome, consistent with the reported cleavage (Fig. 3A-D, Supplementary Fig. 5). In the cryo‑EM maps, density could be traced on both sides of the cleavage site, with no continuous density connecting the two fragments. In contrast, the equivalent region in the mouse ribosome has continuous but fragmented density. This lower local resolution limits the precision of rRNA modeling but still supports an intact 28S rRNA configuration. This comparison highlights the presence of a structurally well-resolved rRNA discontinuity in the naked mole-rat ribosome, contrasted with the intact but flexible corresponding region in the mouse.

Local resolution maps of the NMR ribosome showed uniform resolution and suggested that the discontinuity in the NMR ribosome is a feature, not a technical artefact. The map density is well-resolved on both flanks of the gap, and the region as a whole is of high resolution (Fig. 3H, Supplementary Fig. 5). The missing density is therefore a true rRNA break, unlike in the mouse ribosome, where this region is intact but flexible. Mapping the local environment around the cleavage site showed that several ribosomal proteins (uL4, eL6, eL18, eL28, and uL30) are positioned to support the two rRNA fragments (Fig. 3E). The proteins at this site are positioned similarly in both the naked mole-rat and mouse ribosomes, with RMSDs below 1.5 Å (Fig. 3F), and this arrangement is likewise preserved when compared to the high-resolution human structure. These proteins likely stabilize the break through a network of interactions, helping to maintain the integrity of the large subunit despite the discontinuity. A 28S rRNA fragment at the cleavage site and all proteins within a 12 Å radius were extracted for PDBePISA analysis (Fig. 3G). Calculations of the buried surface area and solvation free energy at the interface showed that the degree of protein-RNA contacts and the energetic favorability of the interface are comparable across NMR, mouse, and human (Fig. 3I). This analysis demonstrates that the protein-rRNA interface is not energetically compromised by the cleavage, providing evidence for a fully compensated and stable architecture of the ribosome.

### The naked mole-rat ribosome maintains a conserved pattern of rRNA modifications and magnesium ion coordination

**Fig. 4:**
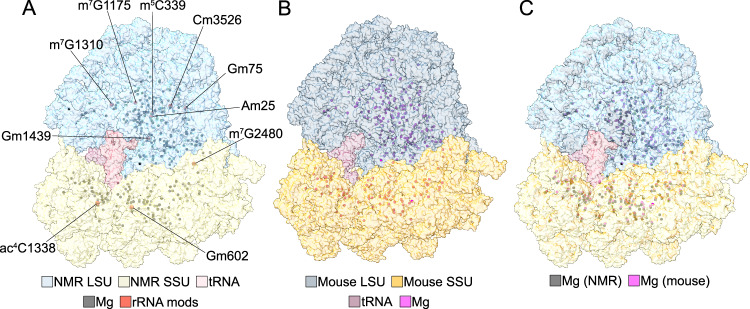
rRNA modifications and Mg²⁺ ion distribution. **A** rRNA modifications and Mg ion distribution in the naked mole-rat ribosome. **B** Mg ion distribution in the mouse ribosome. **C** Superposition of the NMR and mouse ribosomes, comparing the distribution of Mg ions.

Eukaryotic rRNAs are decorated with chemical modifications, primarily 2’-O-methylations (Nm) and pseudouridylations (Ψ), which play crucial roles in ribosome assembly and the fine-tuning of translational function^7,9,20,21^. The high quality of our cryo-EM maps enabled a systematic survey of the rRNA modification landscape in the NMR ribosome. By inspecting the density for features exceeding the standard RNA chemistry, we identified a total of 10 putative modification sites across the 18S, 5.8S, and 28S rRNAs (Fig. 4A, Supplementary Fig. 6, Table 2). These modifications, found in conserved functional regions, reflect the epitranscriptomic signatures previously observed in mammalian ribosomes^22^.

**Table 2:** Putative rRNA Modifications Identified in the Naked Mole-Rat Ribosome

rRNA	Position	Modification	Functional Region
28S	25	Am	Solvent-exposed periphery
28S	339	m5C	Solvent-exposed periphery
28S	1175	m7G	Near the exit tunnel
28S	1310	m7G	Near the E-site
28S	1439	Gm	Near the E-site
28S	2480	m7G	Near the intersubunit face
28S	3526	Cm	Entry to the exit tunnel
18S	602	Gm	Decoding center
18S	1338	ac4C	Decoding center
5.8S	75	Gm	Near the exit tunnel

The distribution of Mg²⁺ ions in the NMR ribosome showed numerous Mg²⁺ ion clusters within the rRNA core, reflecting their role in stabilizing tertiary structure (Fig. 4A, Supplementary Fig. 7). Comparison with the mouse ribosome^18^ (Fig. 4B) revealed an overall similar pattern of Mg²⁺ binding sites, and superposition of the two species showed only minor shifts in ion positions (Fig. 4C). These observations suggest that the NMR ribosome maintains a conserved network of rRNA modifications and Mg²⁺ coordination sites. Together, these features confirm that despite the break in the 28S rRNA, the ribosome’s core functional centers and overall structural integrity are precisely maintained.

## Discussion

In this study, we present high-resolution cryo-EM structures of the naked mole-rat 80S ribosome, capturing four distinct conformational states of the translation elongation cycle. Our analysis reveals that while the overall architecture, ribosomal protein composition, and epitranscriptomic landscape are highly conserved with other mammals, the NMR ribosome possesses a distinct structural feature: a specific cleavage in the D6 region of the 28S rRNA. These structures provide atomic-level visualization of this “hidden break,” previously identified biochemically^5^, and offer a structural foundation.

Our work documents the discontinuity in the 28S rRNA, which remodels the D6 region. Unlike a site of degradation or structural instability, the cleavage site appears to be a stable, defined feature. The two resulting rRNA fragments are held in place by a conserved network of surrounding ribosomal proteins (uL4, eL6, eL18, eL28, and uL30), which show minimal positional deviation compared to their mouse and human counterparts. Furthermore, our interface analysis suggests that the protein-RNA contacts and the energetic stability of this region are comparable to those in mouse and human ribosomes, which possess an intact rRNA backbone. Our findings are consistent with a recent report by Gutierrez-Vargas and colleagues, who also observed the overall preservation of the ribosome core architecture^23^. The work presented here extends this finding by providing a higher-resolution view of four distinct functional states of the elongation cycle, a quantitative analysis of the energetic stability at the cleavage interface, and a complete map of the conserved rRNA modification landscape, together providing a detailed understanding of the functional integrity of the NMR ribosome.

A hypothesis was that this cleavage might introduce local flexibility or remodel the D6 region, potentially as a mechanism to influence translational fidelity or adapt to stress. However, our comparative structural analyses point to the contrary. Despite the discontinuity in the backbone, the local architecture is well-preserved. The surrounding ribosomal proteins maintain their canonical positions, and the energetic stability of the protein-RNA interface is not compromised. This suggests that the NMR ribosome has evolved to compensate for the break, indicating a strong selective pressure to preserve the native architecture of this region. Therefore, the biological significance of this feature must lie in a more subtle mechanism than major structural rearrangement.

In conclusion, our work provides an atomic-resolution view of the naked mole-rat ribosome, shedding light on the structural nature of its distinct 28S rRNA cleavage. We showed that this break is a stable, integral feature of an otherwise highly conserved ribosome. The structure compensates for the break, maintaining the native architecture and energetic stability of the D6 region. These findings lay the groundwork for future investigations to determine whether this distinct rRNA architecture plays a more subtle role in ribosome biogenesis, turnover, or the extraordinary stress resistance of this species.

## Methods

### Purification of ribosomes from naked mole-rat liver tissue

Tissue collection procedures, specifically the humane harvesting of organs for scientific purposes, were done in accordance with § 4(3) of Germany’s Animal Welfare Act (Tierschutzgesetz). These procedures were recorded under the internal license number N 9001/21 of the Max Delbrück Center for Molecular Medicine, and all internal regulations of the institute’s animal welfare guidelines were strictly complied with.

Liver tissue was dissected from a single 2-year-old male naked mole-rat (*Heterocephalus glaber*) and immediately transferred to a chilled dish on ice. All subsequent steps were carried out under ice-cold conditions. The tissue was washed with ice-cold PBS, chopped into small pieces, and homogenized in lysis buffer (20 mM Tris-HCl, pH 7.4, 10 mM MgCl₂, 150 mM KCl, 2 mM DTT, protease inhibitor cocktail) using glass beads and a Precellys Evolution Touch homogenizer. The homogenate was clarified by centrifugation at 10,000 × *g* for 10 min to remove cell debris. The supernatant was layered onto a 30% sucrose cushion and centrifuged for 2 h at 100,000 × *g*. The resulting pellet was resuspended in a minimal volume of wash buffer (20 mM Tris-HCl, pH 7.4, 10 mM MgCl₂, 150 mM KCl) and further purified by sucrose density gradient centrifugation (25–50% sucrose in 5% increments) for 3 h at 100,000 × *g*. Fractions containing ribosomes were identified by absorbance at 254 nm using a NanoDrop spectrophotometer, pooled, pelleted, and resuspended in sucrose-free buffer (20 mM Tris-HCl, pH 7.4, 10 mM MgCl₂, 150 mM KCl).

### Cryo-EM grid preparation

Cu300 R2/2 grids with an additional 2 nm amorphous carbon support film (Quantifoil Micro Tools) were glow-discharged in a PELCO easiGlow Glow Discharge Cleaning System at 15 mA for 45 s. 4 µl of purified ribosome sample was applied to each grid and incubated for 15 s. Grids were blotted for 4 s at 4 °C with a blot force of 10 and plunge-frozen in liquid ethane cooled to LN2 temperature using a Vitrobot Mark IV (Thermo Fisher Scientific).

### Cryo-EM data acquisition

For data acquisition, frames were collected on a Titan Krios G3i transmission electron microscope (Thermo Fisher Scientific) equipped with a field emission gun, a BioQuantum post-column energy filter (Gatan), and a K3 direct electron detector (Gatan). Movies were recorded at an acceleration voltage of 300 kV in low-dose mode as dose-fractionated videos with a maximum image shift of 5 µm enabled by aberration-free image shift. In total, 16803 movies were collected, each with a total dose of 60.04 e⁻/Å² distributed over 31 fractions (1.94 e⁻/Å² per frame). Data were acquired in energy-filtered zero-loss mode (slit width 20 eV) in nanoprobe mode at a nominal magnification of 105kx, corresponding to a calibrated pixel size of 0.83 Å at the specimen level, using super-resolution mode and a 100 µm objective aperture. Defocus values ranged from −1.0 to −3.0 µm. Data were collected on Quantifoil R2/2 Cu 300 mesh grids, with 8 exposures acquired per hole.

### Cryo-EM image processing

The collected movies were corrected for beam-induced motion using patch alignment and dose-weighted with MotionCor2^24^. Contrast transfer function parameters were estimated with Gctf^25^. Particles were automatically picked using the Laplacian-of-Gaussian algorithm implemented in RELION 4^26^ and extracted with a pixel size of 1.10 Å. Subsequent 2D and 3D classifications were performed in RELION 4, and ribosomal particle subsets were imported into CryoSPARC v4.6.0^27^ for further refinement. Following an initial round of non-uniform refinement, an additional 3D classification step with a focused mask on the decoding and peptidyl transferase centers revealed four distinct conformational states (Supplementary Fig. 1). These classes were refined independently by iterative non-uniform refinement and local refinement of individual subunits. Refinement was concluded once improvements in resolution and map quality became negligible. Final reconstructions were sharpened using standard B-factor correction in CryoSPARC. Local resolutions were estimated in cryoSPARC (FSC = 0.143) for the 80S ribosome and for the cleavage site using a mask encompassing the D6 region; histogram bins were computed from voxel-wise values within this mask.

### Model building and refinement

The previously published atomic model of the mouse 80S ribosome (PDB ID: 7CPU) was used as the initial template. The model was fitted into the cryo-EM density by rigid-body docking in UCSF ChimeraX. The specific NMR sequences were obtained from the mHetGlaV2, pHetGlaV2, mHetGlaV3, and pHetGlaV3 genome assemblies. The homology models were initially created by threading the NMR sequences onto the mouse backbone, followed by manual adjustment in Coot to fit the specific NMR density. RNA and protein residues were removed in regions where the density was absent or highly fragmented. Structured elements were adjusted through iterative cycles of manual model building in Coot v0.9.8.95^28^ and real-space refinement in Phenix v1.21^29^, applying secondary structure restraints throughout. The mRNA was modeled based on the overall density features, as these represent an averaged signal from multiple heterogeneous conformations. Densities corresponding to tRNAs in the A, P, and E sites represent a mixture of endogenous tRNA species. Consequently, atomic models for these regions were adapted from the high-resolution structure of the mouse 80S ribosome (PDB ID: 7CPU). The tRNA models were rigid-body fitted into the cryo-EM density maps using Coot v0.9.8.95, followed by real-space refinement. Given the averaging of variable regions in the map, the mouse tRNA sequence was maintained as a representative structural scaffold. The identification of the Mg^2+^ ions was based on three criteria: (1) strong spherical density, (2) appropriate distances to phosphate oxygens (~2.0–2.2 Å), and (3) structural conservation with high-resolution mouse and human ribosome structures. The final models were validated using MolProbity, yielding favorable statistics for both states (Table S1).

### Assignment of rRNA modifications

Putative rRNA modifications were assigned by visual inspection of the cryo-EM density. Each nucleotide was examined for additional density features at the nucleobase and the 2′-OH group of the ribose sugar. Residues displaying such features were modeled as modified nucleotides in Coot. Given the local variation in map resolution, each potential modification site was evaluated individually by adjusting the map contour level to ensure consistency with the surrounding density.

### Statistical analysis

No statistical methods were used to predetermine sample size. The cryo-EM data processing workflow, including particle numbers for each class, is detailed in Supplementary Fig. 1.

### Figure generation

All figures showing structural models were generated using UCSF ChimeraX.

### Reporting summary

Further information on research design is available in the Nature Portfolio Reporting Summary linked to this article.

## Supplementary information

Supplementary Information

Reporting Summary

Transparent Peer Review file

## Data Availability

The raw cryo-ET data have been deposited to the EM Public Image Archive under accession code EMPIAR-13350. Maps have been deposited in the EMDB under accession codes EMD-55098, EMD-55100, EMD-56709, and EMD-56710. Atomic models have been deposited in the PDB under accession codes 9T5B, 9T5C, 28PI, and 28PJ.
