# Supplementary material for: Cryo-EM structure of the naked mole-rat ribosome reveals a stabilized split 28S rRNA
Source: Nat Commun. 2026 Jul 3;17:5841. doi: 10.1038/s41467-026-75143-0 (PMC13332236; doi:10.1038/s41467-026-75143-0)
Supplement: Supplementary file 1 — Supplementary Information [file 41467_2026_75143_MOESM1_ESM.pdf]

Supplementary Material for

Cryo-EM structure of the naked mole-rat ribosome reveals a stabilized split 28S rRNA

Mehmet Gül[1,2], Alice Rossi[3], Christian M.T. Spahn[4], Gary R. Lewin[3,5], Mikhail Kudryashev[1,4,*]

[1] *In situ* Structural Biology, Max Delbrück Center for Molecular Medicine in the Helmholtz Association (MDC), 13125 Berlin, Germany

[2] Institute for Chemistry and Biochemistry, Freie Universität Berlin, 14195 Berlin, Germany

[3] Molecular Physiology of Somatic Sensation Laboratory, Max Delbrück Center for Molecular Medicine in the Helmholtz Association (MDC), 13125 Berlin, Germany

[4] Charité – Universitätsmedizin Berlin, corporate member of Freie Universität Berlin and Humboldt Universität zu Berlin, Institute for Medical Physics and Biophysics, Berlin, Germany

[5.] Charité – Universitätsmedizin Berlin, corporate member of Freie Universität Berlin and Humboldt Universität zu Berlin, Berlin, Germany

* Correspondence to Mikhail.Kudryashev@mdc-berlin.de

This file contains:

Supplementary Figures 1, 2, 3, 4, 5, 6, 7

Supplementary Table 1

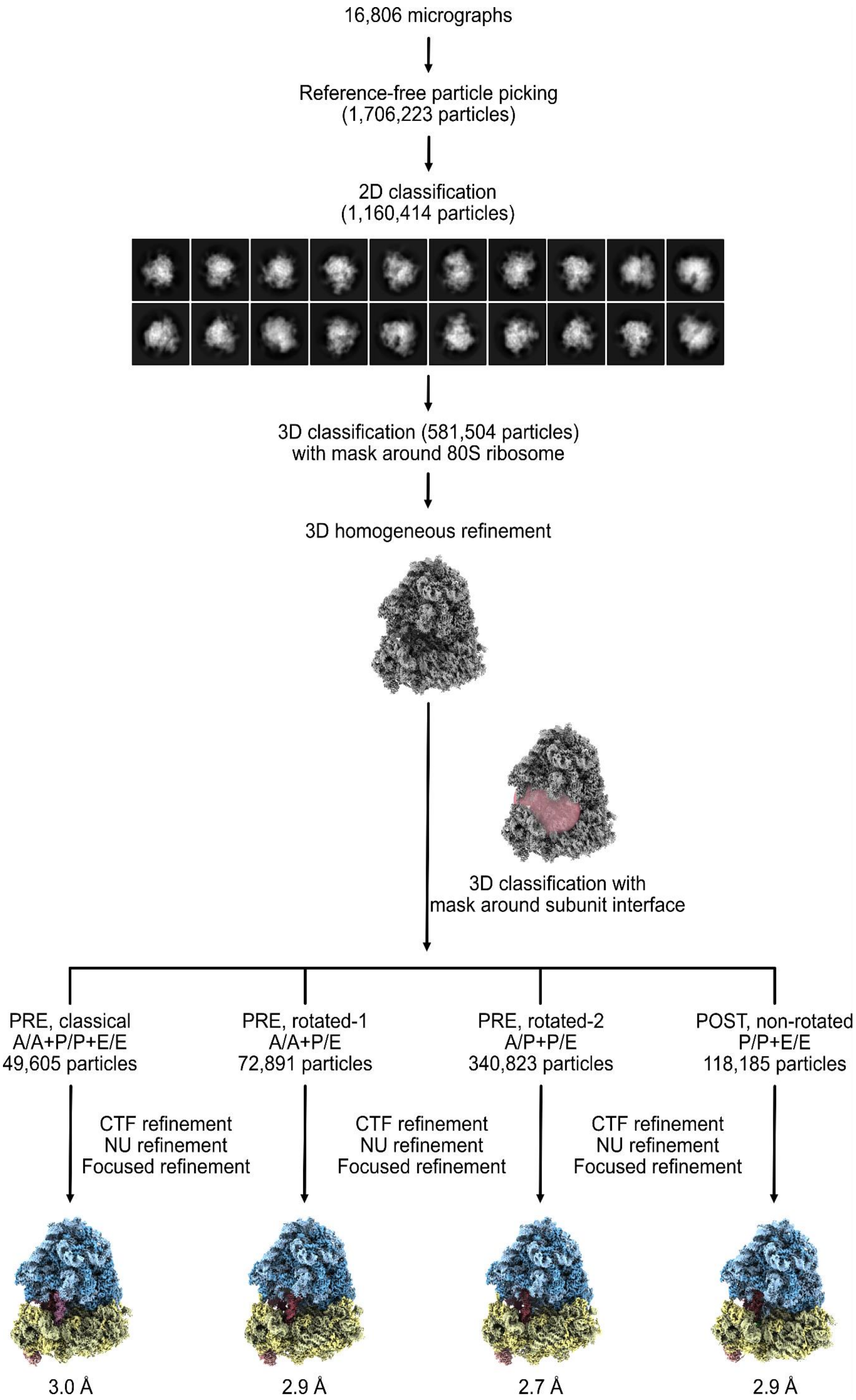

Supplementary Fig. 1: Cryo-EM data processing workflow. Reference-free particle

picking from 16,806 micrographs yielded 1,706,223 particles, which were reduced to 1,160,414 particles after 2D classification (representative class averages shown). 3D classification with a mask around the 80S ribosome selected 581,504 particles for 3D homogeneous refinement, producing a consensus map of the full ribosome. A second round of 3D classification, using a mask focused on the subunit interface (highlighted in pink), separated particles into four elongation states based on tRNA and ribosomal subunit configuration. Final maps are colored by ribosomal component (40S, yellow; 60S, blue; tRNAs/RACK1, pink).

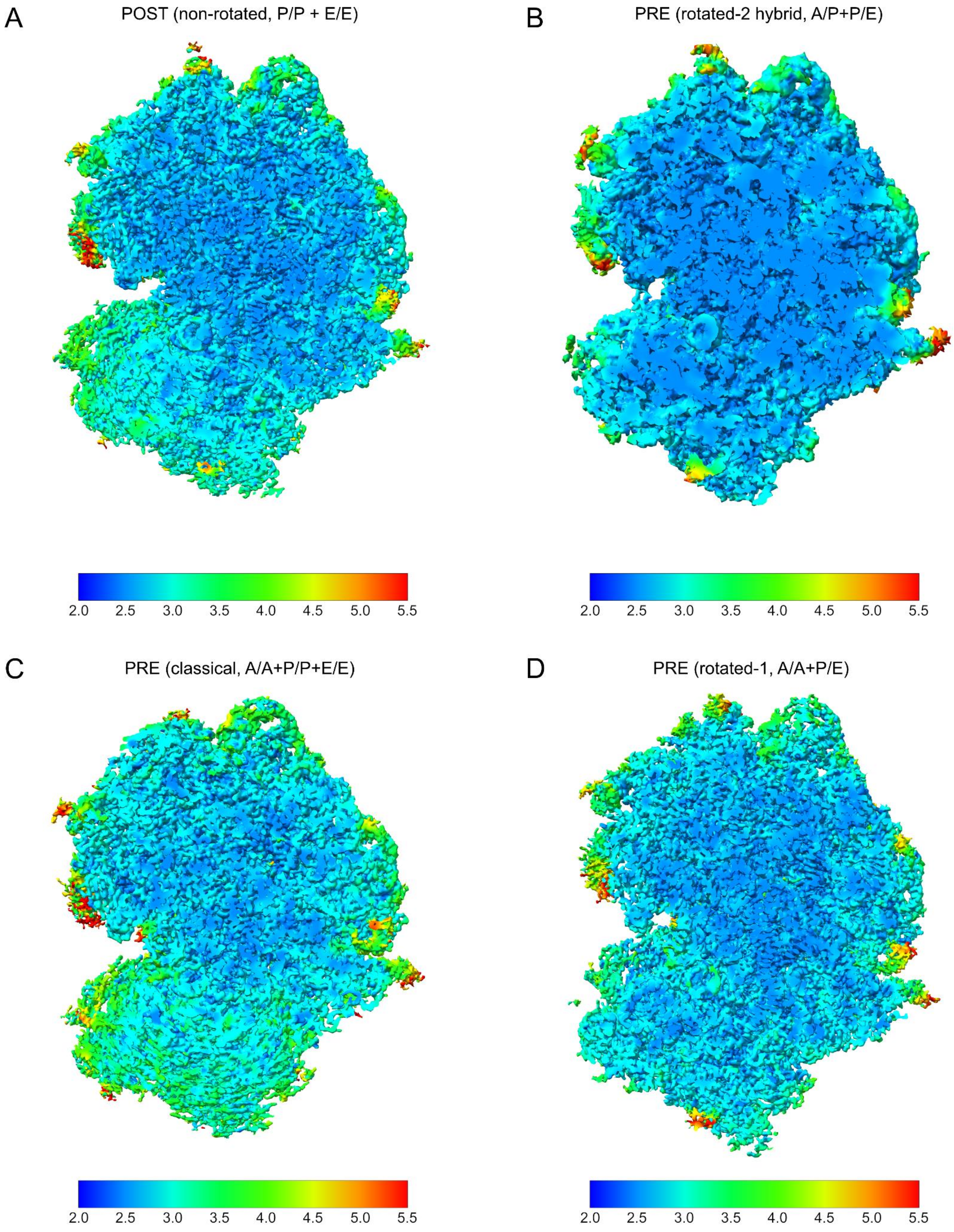

Supplementary Fig. 2: **Cutaway view through the cryo-EM density maps** of the (A) POST, (B) rotated-2 PRE, (C) classical PRE, and (D) rotated-1 PRE states. The maps are colored by the local resolution as calculated in cryoSPARC.

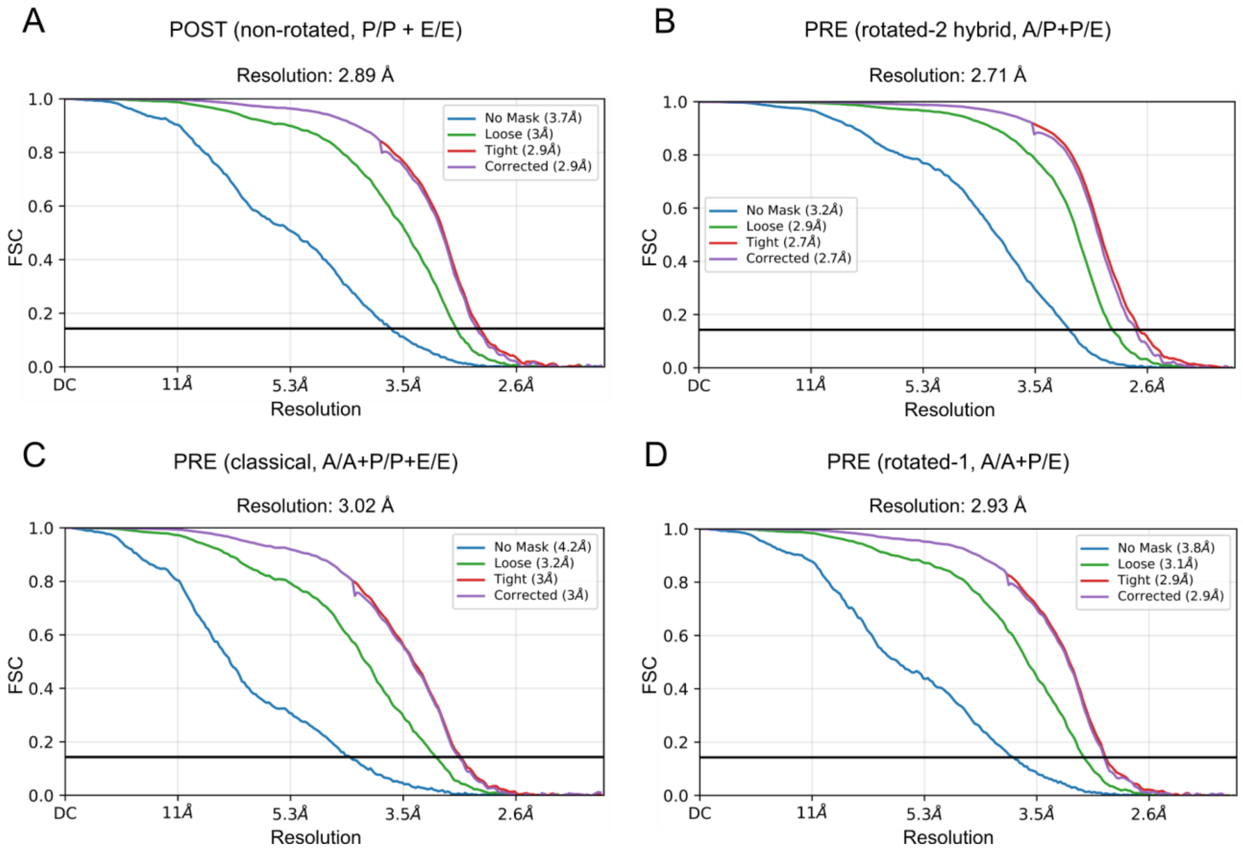

Supplementary Fig. 3: **Resolution of structures measured by Fourier Shell Correlation (FSC)** for the (A) POST, (B) rotated-2 PRE, (C) classical PRE, and (D) rotated-1 PRE states. The resolution values are shown for the cutoff value of 0.143.

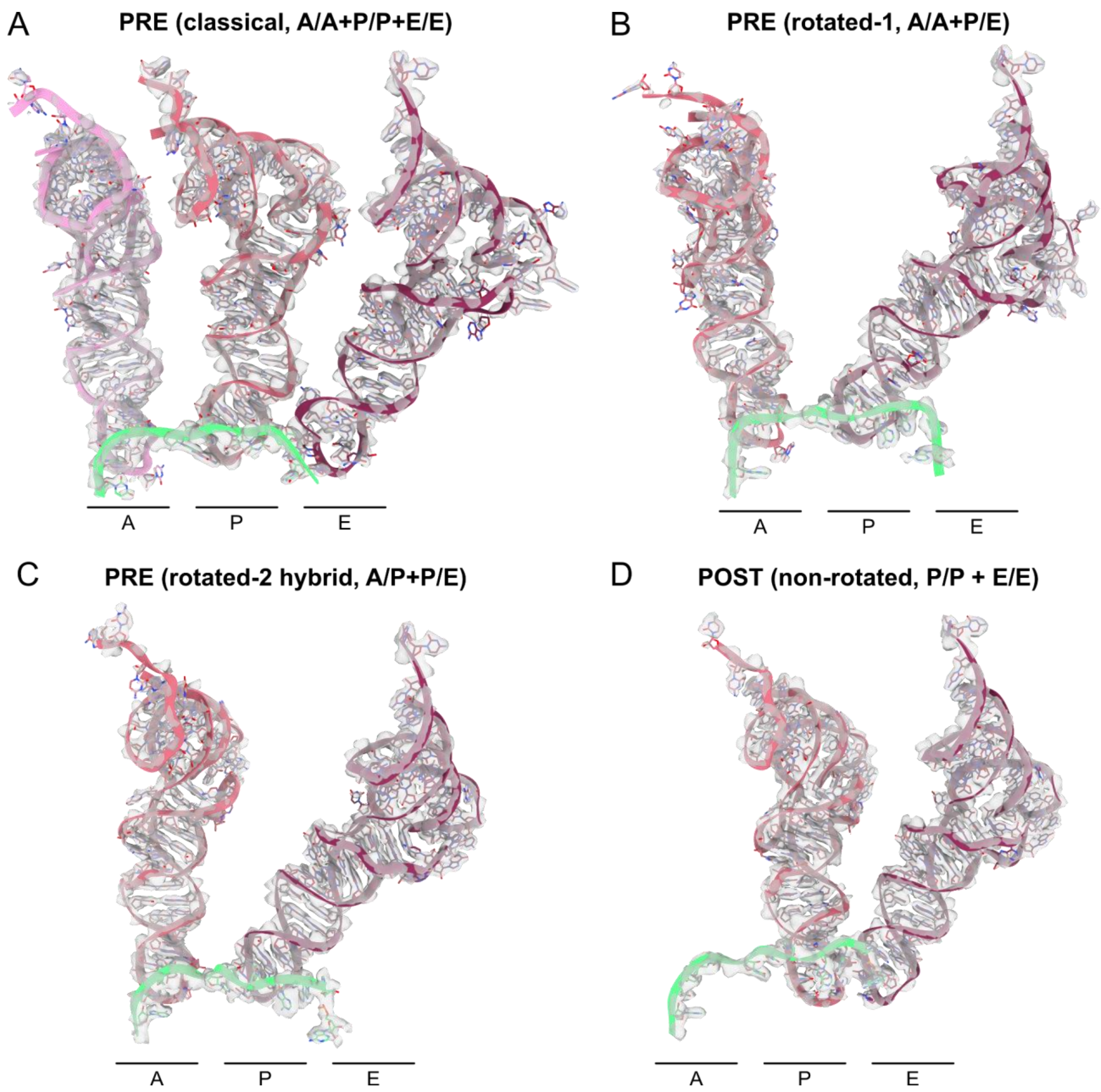

Supplementary Fig. 4: **Close-up densities with fitted tRNA models.** (A) A/A, P/P, E/E tRNAs in classical PRE, (B) A/A, P/E tRNAs in rotated-1 PRE, (C) A/P, P/E tRNAs in rotated-2 PRE, and (D) P/P, E/E tRNAs in POST states.

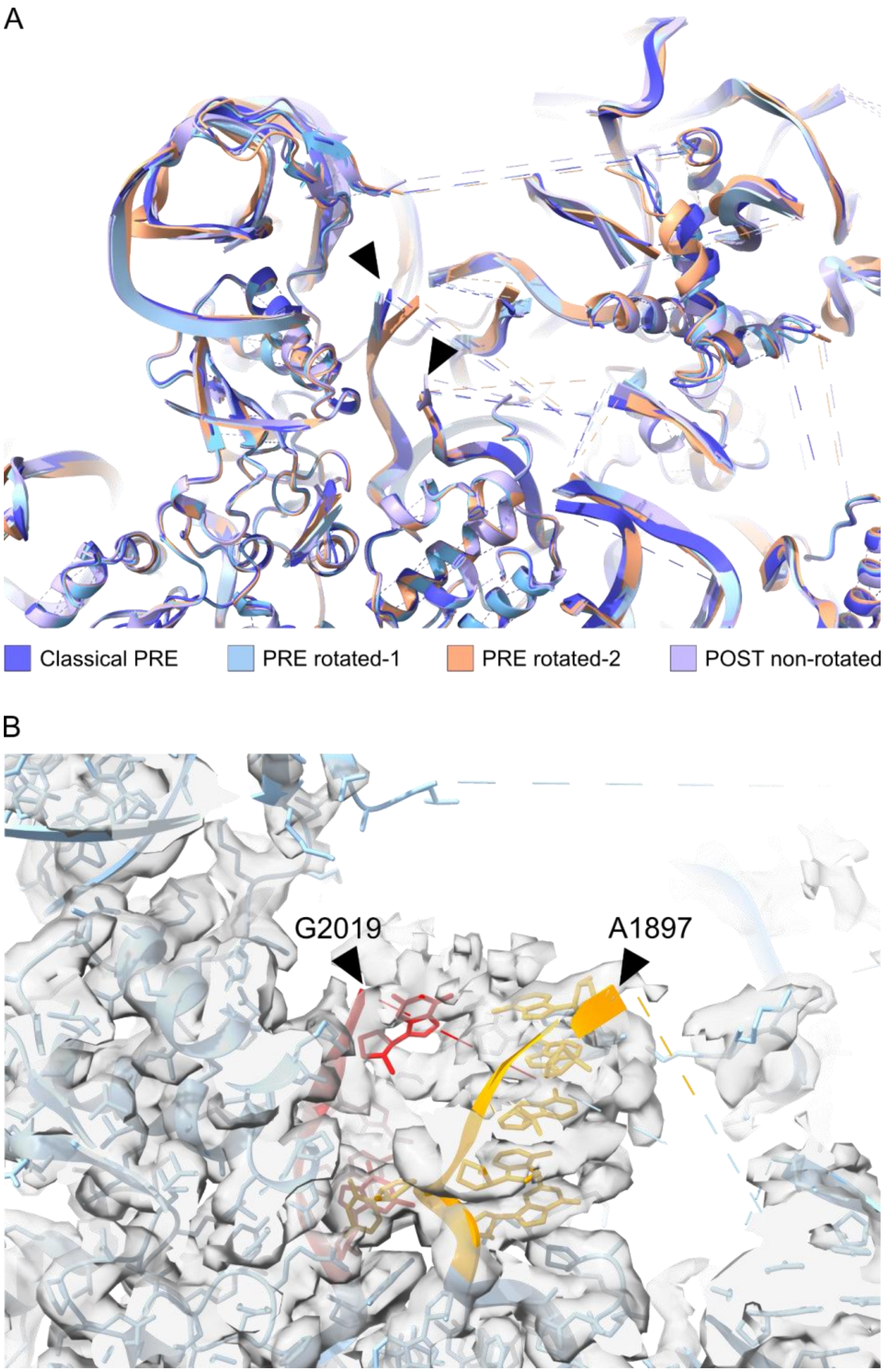

Supplementary Fig. 5: **28S rRNA cleavage site.** (A) Superposition of the atomic models of the four states identified. Color coding for the models is shown in the legend below. (B) Close-up density of the cleavage site with a fitted model (PRE rotated-2). The rRNA break points are indicated with black arrowheads and residue numbers. The flanking rRNA fragments are shown in yellow and red for clarity.

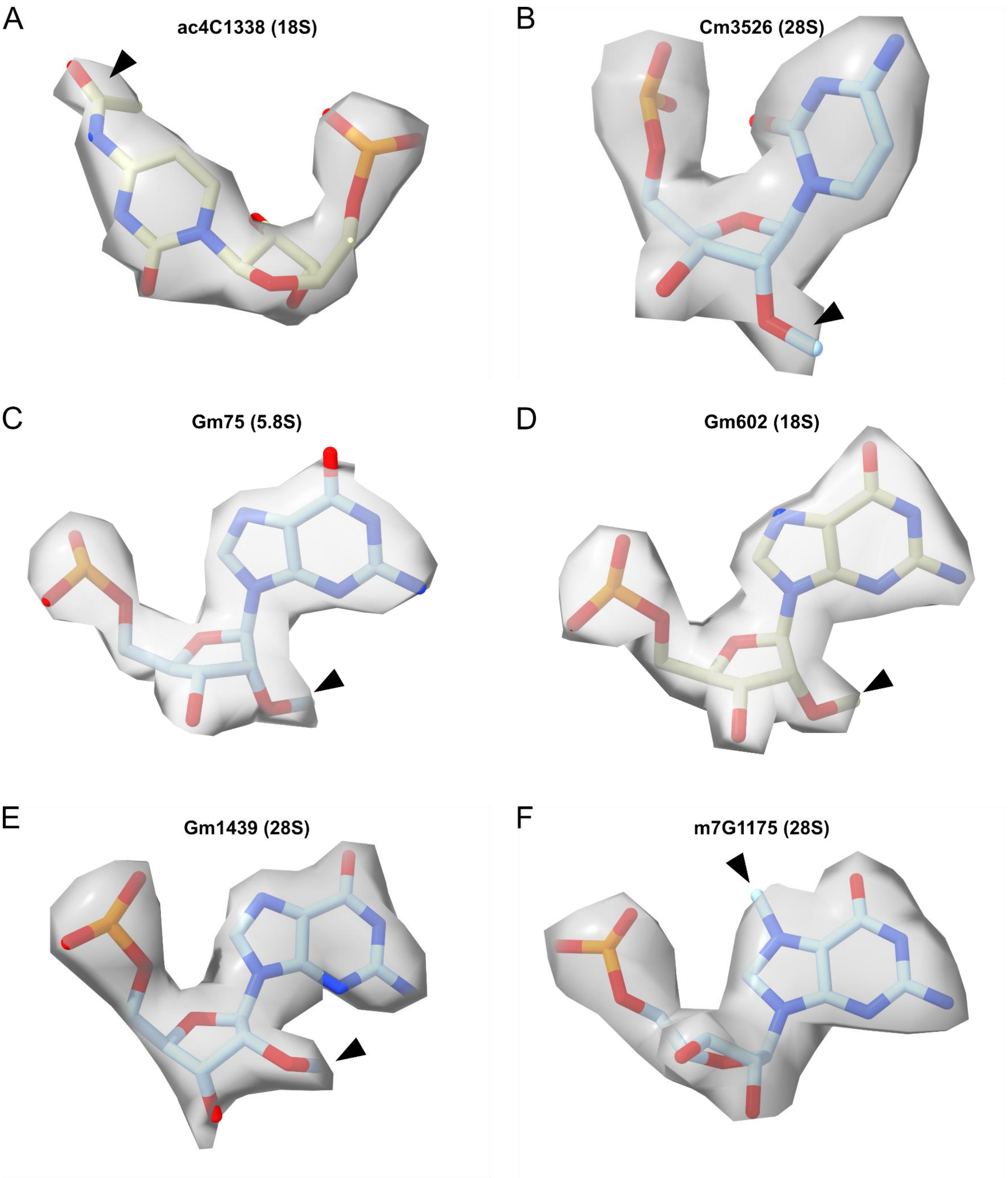

Supplementary Fig. 6: Close-up densities with fitted models for rRNA modifications. (A) ac4C1338 in 18S rRNA, (B) Cm3526 in 28S rRNA, (C) Gm75 in 5.8S rRNA, (D) Gm602 in 18S rRNA, (E) Gm1439 in 28S rRNA, (F) m7G1175 in 28S rRNA. The modification sites are indicated by black arrowheads. The rRNA chains are written in parentheses. The models are colored by atoms (light blue/light yellow, carbon; dark blue, nitrogen; red, oxygen).

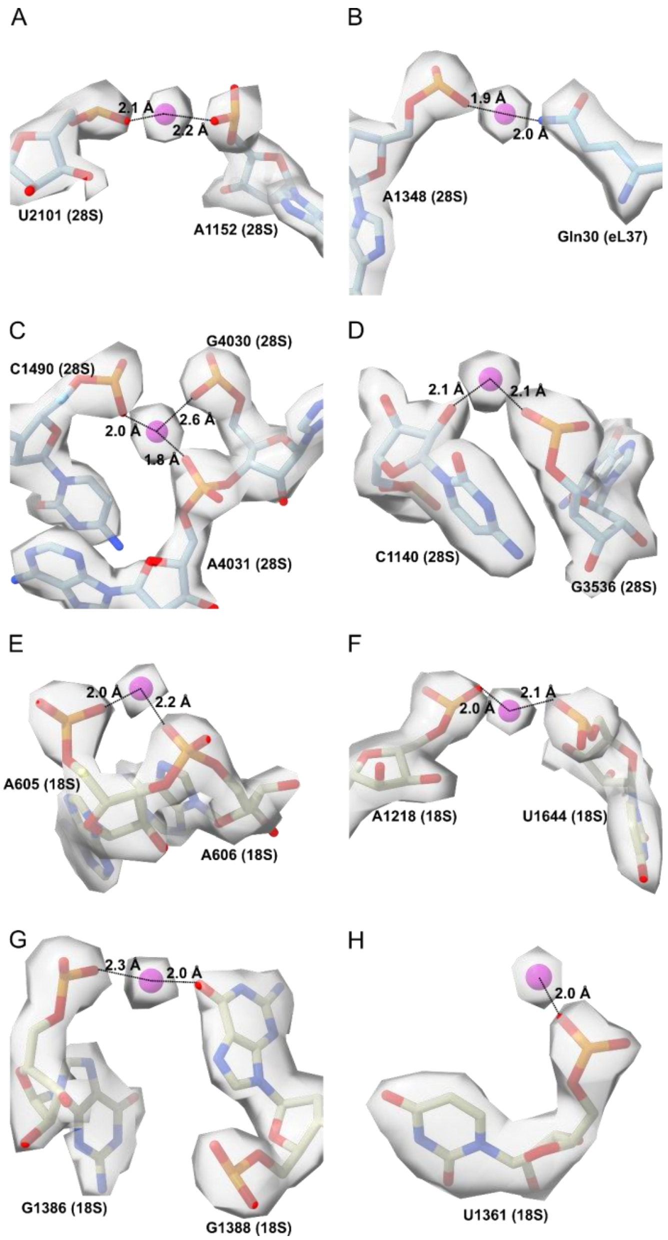

Supplementary Fig. 7: Close-up densities with fitted models for the Mg^{2+} ions and their coordination shells. Mg^{2+} ion between (A) A1152 and U2101 of 28S rRNA, (B) A1348 of 28S rRNA and Gln30 of ribosomal protein eL37, (C) C1490, G4030 and A4031 of 28S rRNA, (D) C1140 and G3536 of 28S rRNA, (E) A605 and A606 of 18S rRNA, (F) A1218 and U1644 of 18S rRNA, (G) G1386 and G1388 of 18S rRNA, and (H) with U1361 of 18S rRNA. The Mg^{2+} ions are shown as magenta spheres, while the nucleotides and amino acids are shown in stick representation. The models are colored by atoms (light blue/light yellow, carbon; dark blue, nitrogen; red, oxygen; magenta, magnesium)

Supplementary Table 1: Cryo-EM Data Collection, Refinement, and Validation Statistics

	POST	PRE rotated-2	Classical PRE	PRE rotated-1
**Data Collection and Processing**				
Microscope	Titan Krios G3i	Titan Krios G3i	Titan Krios G3i	Titan Krios G3i
Detector	Gatan K3 + Bioquantum GIF	Gatan K3+ Bioquantum GIF	Gatan K3+ Bioquantum GIF	Gatan K3+ Bioquantum GIF
Voltage (kV)	300	300	300	300
Electron dose (e^{-}/A^{2})	60.04	60.04	60.04	60.04
Defocus range (μm)	-1.0 to -3.0	-1.0 to -3.0	-1.0 to -3.0	-1.0 to -3.0
Pixel size (Å)	0.83	0.83	0.83	0.83
Initial particles	1706223	1706223	1706223	1706223
Final particles	118185	340823	49605	72891
Map resolution (Å)	2.9	2.7	3.0	2.9
Map sharpening B-factor (A^{2})	-68.79	-82.94	-80.40	-89.40
**Model Refinement**				
Initial model used (PDB ID)	7CPU	7CPU	7CPU	7CPU
Model components	72 proteins, 4 rRNAs	72 proteins, 4 rRNAs	72 proteins, 4 rRNAs	72 proteins, 4 rRNAs
Map-model FSC (FSC=0.5)	3.0	2.8	3.19	3.05
Correlation Coefficient (CC_mask)	0.87	0.86	0.82	0.84
**Model Validation (MolProbity)**				
MolProbity score	1.66	1.63	1.83	1.42
Clashscore	5.32	3.50	7.32	2.89
Ramachandran favored	94.68	91.83	93.56	95.05

(%)				
Ramachandran allowed (%)	5.22	7.21	6.29	4.80
Ramachandran outliers (%)	0.10	0.96	0.15	0.15
Rotamer outliers (%)	1.02	0.62	0.80	0.36
RMSD bonds (Å)	0.004	0.005	0.006	0.003
RMSD angles (°)	0.573	0.733	0.845	0.555
