# Supplementary material for: Cryo-EM structure of the naked mole-rat ribosome reveals a stabilized split 28S rRNA
Source: Nat Commun. 2026 Jul 3;17:5841. doi: 10.1038/s41467-026-75143-0 (PMC13332236; doi:10.1038/s41467-026-75143-0)
Supplement: Supplementary file 2 — Reporting Summary [file 41467_2026_75143_MOESM2_ESM.pdf]

nature portfolio

Corresponding author(s): Mikhail Kudryashev

Last updated by author(s): 2026/06/12

Reporting Summary

Nature Portfolio wishes to improve the reproducibility of the work that we publish. This form provides structure for consistency and transparency in reporting. For further information on Nature Portfolio policies, see our Editorial Policies and the Editorial Policy Checklist.

Statistics

For all statistical analyses, confirm that the following items are present in the figure legend, table legend, main text, or Methods section.

n/a	Confirmed	
☒	☐	The exact sample size (*n*) for each experimental group/condition, given as a discrete number and unit of measurement
☒	☐	A statement on whether measurements were taken from distinct samples or whether the same sample was measured repeatedly
☒	☐	The statistical test(s) used AND whether they are one- or two-sided<br>*Only common tests should be described solely by name; describe more complex techniques in the Methods section.*
☒	☐	A description of all covariates tested
☒	☐	A description of any assumptions or corrections, such as tests of normality and adjustment for multiple comparisons
☒	☐	A full description of the statistical parameters including central tendency (e.g. means) or other basic estimates (e.g. regression coefficient) AND variation (e.g. standard deviation) or associated estimates of uncertainty (e.g. confidence intervals)
☒	☐	For null hypothesis testing, the test statistic (e.g. *F*, *t*, *r*) with confidence intervals, effect sizes, degrees of freedom and *P* value noted<br>*Give P values as exact values whenever suitable.*
☒	☐	For Bayesian analysis, information on the choice of priors and Markov chain Monte Carlo settings
☒	☐	For hierarchical and complex designs, identification of the appropriate level for tests and full reporting of outcomes
☒	☐	Estimates of effect sizes (e.g. Cohen's *d*, Pearson's *r*), indicating how they were calculated

Our web collection on statistics for biologists contains articles on many of the points above.

Software and code

Policy information about availability of computer code

Data collection	Thermo Fisher Scientific EPU version 3.11
Data analysis	RELION 4.0.2, CryoSPARC v4.6.0, ChimeraX 1.9, Phenix v1.21, Coot v0.9.8.95

For manuscripts utilizing custom algorithms or software that are central to the research but not yet described in published literature, software must be made available to editors and reviewers. We strongly encourage code deposition in a community repository (e.g. GitHub). See the Nature Portfolio guidelines for submitting code & software for further information.

Data

Policy information about availability of data

All manuscripts must include a data availability statement. This statement should provide the following information, where applicable:

- Accession codes, unique identifiers, or web links for publicly available datasets
- A description of any restrictions on data availability
- For clinical datasets or third party data, please ensure that the statement adheres to our policy

The raw cryo-ET data have been deposited to the EM Public Image Archive under accession code EMPIAR-13350. Maps have been deposited in the EMDB under accession codes EMD-55098 [https://www.ebi.ac.uk/emdb/EMD-55098], EMD-55100 [https://www.ebi.ac.uk/emdb/EMD-55100], EMD-56709 [https://www.ebi.ac.uk/emdb/EMD-56709], and EMD-56710 [https://www.ebi.ac.uk/emdb/EMD-56710]. Atomic models have been deposited in the PDB under accession

codes 9T5B [https://doi.org/10.2210/pdb9t5b/pdb], 9T5C [https://doi.org/10.2210/pdb9t5c/pdb], 28PI [https://doi.org/10.2210/pdb28pi/pdb], and 28PJ [https://doi.org/10.2210/pdb28pj/pdb].

Research involving human participants, their data, or biological material

Policy information about studies with human participants or human data. See also policy information about sex, gender (identity/presentation), and sexual orientation and race, ethnicity and racism.

Reporting on sex and gender	NA
Reporting on race, ethnicity, or other socially relevant groupings	*Please specify the socially constructed or socially relevant categorization variable(s) used in your manuscript and explain why they were used. Please note that such variables should not be used as proxies for other socially constructed/relevant variables (for example, race or ethnicity should not be used as a proxy for socioeconomic status).*<br>*Provide clear definitions of the relevant terms used, how they were provided (by the participants/respondents, the researchers, or third parties), and the method(s) used to classify people into the different categories (e.g. self-report, census or administrative data, social media data, etc.)*<br>*Please provide details about how you controlled for confounding variables in your analyses.*
Population characteristics	*Describe the covariate-relevant population characteristics of the human research participants (e.g. age, genotypic information, past and current diagnosis and treatment categories). If you filled out the behavioural & social sciences study design questions and have nothing to add here, write "See above."*
Recruitment	*Describe how participants were recruited. Outline any potential self-selection bias or other biases that may be present and how these are likely to impact results.*
Ethics oversight	*Identify the organization(s) that approved the study protocol.*

Note that full information on the approval of the study protocol must also be provided in the manuscript.

Field-specific reporting

Please select the one below that is the best fit for your research. If you are not sure, read the appropriate sections before making your selection.

☒ Life sciences ☐ Behavioural & social sciences ☐ Ecological, evolutionary & environmental sciences

For a reference copy of the document with all sections, see nature.com/documents/nr-reporting-summary-flat.pdf

Life sciences study design

All studies must disclose on these points even when the disclosure is negative.

Sample size	No statistical methods were used to predetermine sample size. A total of 1,706,223 particles were initially picked from 16,803 micrographs. After 2D and 3D classification, 49,605–340,823 particles were retained per conformational state, which is consistent with established practice for high-resolution single-particle cryo-EM of ribosome complexes.
Data exclusions	Particles were excluded during iterative 2D and 3D classification in RELION 4 and cryoSPARC based on poor class averages or failure to classify into interpretable conformational states. Exclusion criteria were based on standard image quality metrics inherent to the software and were not pre-established prior to data collection.
Replication	The structural findings are based on a single cryo-EM dataset collected from one ribosome preparation. The four conformational states were independently refined and each converged to consistent, high-resolution reconstructions. Reproducibility is supported by the internal consistency of the maps, map-model FSC values, and agreement with published mammalian ribosome structures.
Randomization	Randomization is not applicable to this study. No experimental groups were defined; all ribosomes were purified from a single species and subjected to unbiased computational classification.
Blinding	Blinding was not applicable. This is a structural study with no group allocation. Conformational states were assigned computationally based on objective classification criteria without investigator intervention.

Reporting for specific materials, systems and methods

We require information from authors about some types of materials, experimental systems and methods used in many studies. Here, indicate whether each material, system or method listed is relevant to your study. If you are not sure if a list item applies to your research, read the appropriate section before selecting a response.

Materials & experimental systems

n/a	Involved in the study
☒	☐ Antibodies
☒	☐ Eukaryotic cell lines
☒	☐ Palaeontology and archaeology
☐	☒ Animals and other organisms
☒	☐ Clinical data
☒	☐ Dual use research of concern
☒	☐ Plants

Methods

n/a	Involved in the study
☒	☐ ChIP-seq
☒	☐ Flow cytometry
☒	☐ MRI-based neuroimaging

Animals and other research organisms

Policy information about studies involving animals; ARRIVE guidelines recommended for reporting animal research, and Sex and Gender in Research

Laboratory animals	Naked mole-rat (Heterocephalus glaber), wild-type, age: 2y 6mo
Wild animals	The study did not involve wild animals.
Reporting on sex	This information has not been collected.
Field-collected samples	The study did not involve samples collected from the field.
Ethics oversight	All experimental procedures and housing conditions were approved by the Berlin Animal Ethics Committee (Landesamt für Gesundheit und Soziales, Berlin, License: N9001/26) and conducted in accordance with the European Union Directive 2010/63/EU on the protection of animals used for scientific purposes.

Note that full information on the approval of the study protocol must also be provided in the manuscript.

Plants

Seed stocks	*Report on the source of all seed stocks or other plant material used. If applicable, state the seed stock centre and catalogue number. If plant specimens were collected from the field, describe the collection location, date and sampling procedures.*
Novel plant genotypes	*Describe the methods by which all novel plant genotypes were produced. This includes those generated by transgenic approaches, gene editing, chemical/radiation-based mutagenesis and hybridization. For transgenic lines, describe the transformation method, the number of independent lines analyzed and the generation upon which experiments were performed. For gene-edited lines, describe the editor used, the endogenous sequence targeted for editing, the targeting guide RNA sequence (if applicable) and how the editor was applied.*
Authentication	*Describe any authentication procedures for each seed stock used or novel genotype generated. Describe any experiments used to assess the effect of a mutation and, where applicable, how potential secondary effects (e.g. second site T-DNA insertions, mosiacism, off-target gene editing) were examined.*
