# Supplementary material for: Cryo-EM structure of the naked mole-rat ribosome reveals a stabilized split 28S rRNA
Source: Nat Commun. 2026 Jul 3;17:5841. doi: 10.1038/s41467-026-75143-0 (PMC13332236; doi:10.1038/s41467-026-75143-0)
Supplement: Supplementary file 3 — Transparent Peer Review file [file 41467_2026_75143_MOESM3_ESM.pdf]

nature portfolio

Peer Review File

Cryo-EM structure of the naked mole-rat ribosome reveals a stabilized split 28S rRNA

Corresponding Author: Professor Mikhail Kudryashev

This file contains all reviewer reports in order by version, followed by all author rebuttals in order by version.

Version 0:

Reviewer comments:

Reviewer #1

(Remarks to the Author)
The manuscript by Gul and colleagues reports two cryo-EM structures of the ribosome purified from naked mole rat (NMR) liver. The rationale for this work is that while NMR is a popular animal model in the aging, cancer and other research fields, ribosome structures from this organism have not been reported. Furthermore, the large rRNA is fragmented; this unusual feature, in comparison to other mammals, may result in a peculiarity in the ribosome structure with functional implications. The authors describe two elongation states at 2.7 to 2.9 A average resolution, bound with two tRNAs within a rotated or non-rotated ribosome state. The structures are similar to mouse and human ribosomes, as expected from high sequence conservation. Despite being fragmented, the 28S rRNA and the proteins surrounding the cleavage site are structured similarly to those in non-fragmented homologs, in keeping with high structure conservation. The authors also describe several rRNA modified nucleotides and assign Mg+2 ions coordinated by rRNA. The quality of the cryo-EM data and maps appears high, although additional illustrations are needed to support main findings (see below). As the first report of NMR ribosomes, this study will be a valuable resource.
1. It is somewhat surprising that particle classification resulted in two clean classes. In most studies, multiple ribosome states commonly arise, with different tRNA and translation factor occupancies. The two classes may therefore have sub-stoichiometric tRNAs or mixtures of other states with low-occupancy factors (i.e., poorly visible at high contour levels). Have the authors tried classifications into multiple classes or using masks in different locations, such as the GTPase center? The latter may reveal minor classes with translation factors.
2. In the classification figure (S2), add the position of the mask (e.g., in the starting reconstruction).
3. The key features emphasized in the manuscript are not illustrated as close-up density views with fitted structures. Support these conclusions by corresponding close-up views:
a. the rRNA cleavage site
b. rRNA modifications
c. Mg2+ coordination sites
4. How the ion densities were unambiguously annotated as Mg2+ (as opposed to other ions)? Describe in Methods and show a few examples of densities with coordination geometry, as noted above.
5. A mouse ribosome structure was used as a starting model; explain in Methods how the NMR sequences for rRNA and proteins were traced, and which sequences were used for reference.
6. Due to compositional heterogeneity, which tRNA species were chosen to fit best into densities? Explain in Methods, and provide model-density close-up views.
7. In the data/model statistics table, report the CCs between structural models and maps.

Andrei Korostelev

Reviewer #2

(Remarks to the Author)
The manuscript by Gul and coworders reports two cryo-EM structures of the naked mole-rat (NMR, Heterocephalus glaber) cytosolic ribosome captured in two elongation states: a pre-translocation state (80S–A/P- and P/E-tRNAs) and a post-translocation state (80S–P- and E-tRNAs). The NMR is of particular interest due to its exceptional lifespan, resistance to age-related diseases, and tolerance to various stress conditions. Previous work (Azpurua et al., 2013) demonstrated that the

28S rRNA in NMR is cleaved into two fragments via two cuts within the D6 expansion segment, excising a ~260 nt fragment. That study also showed that although NMR fibroblasts exhibit translation rates comparable to mouse fibroblasts, their translation fidelity is significantly higher, yet this increase could not be directly attributed to the unique 28S rRNA cleavage. Nonetheless, both the unusual rRNA processing and elevated translational fidelity are attractive underlying features that offer unique biology to this organism. The current manuscript aims to characterize the structural integrity of NMR ribosomes harboring the cleaved 28S rRNA and to address how this feature might influence translation fidelity. The authors' main observations and conclusions can be summarized as follows:
1. The conformational changes observed between the two cryo-EM structures are consistent with the canonical elongation cycle described for other eukaryotic ribosomes.
2. The overall architecture of the NMR ribosome is highly similar to that of mouse and human cytosolic ribosomes.
3. Despite the cleavage in the 28S rRNA, the local environment surrounding the break, including the positions of neighboring ribosomal proteins uL4, eL6, eL18, eL28, and uL30, is essentially indistinguishable from that in the mouse ribosome with intact 28S rRNA. Thus, the 28S rRNA cleavage does not appear to induce detectable structural rearrangements in its immediate neighborhood.

Overall, the study provides a structural "snapshot" of the previously described 28S rRNA break within the context of intact 80S ribosomes, thereby confirming that the fragmentation is compatible with a structurally canonical translational apparatus. However, beyond visualizing this rRNA cleavage in situ, the work has limited impact on our mechanistic understanding of how this feature might influence translation fidelity, stress resistance, or other unique physiological traits of the NMR. Further structural analyses of the NMR ribosomes bound to cognate and near-cognate tRNAs, analogous to the work of Loveland et al., (2017), could directly test whether rRNA fragmentation contributes to the reported increase in translational fidelity.

In its current form, the manuscript essentially confirms that (i) the NMR ribosomes are structurally very similar to those of mouse and human, and (ii) the 28S rRNA break does not cause any obvious local structural perturbations. While these are useful descriptive observations, they provide only a modest advance beyond the earlier biochemical characterization and do not yet substantively address the biological significance of the 28S rRNA fragmentation.

Reviewer #3

(Remarks to the Author)
I co-reviewed this manuscript with one of the reviewers who provided the listed reports. This is part of the Nature Communications initiative to facilitate training in peer review and to provide appropriate recognition for Early Career Researchers who co-review manuscripts.

Version 1:

Reviewer comments:

Reviewer #1

(Remarks to the Author)
In this revised manuscript, additional analyses and updated text and figures address most of the concerns I had raised. The following suggestions address the remaining minor manuscript concerns and a structure quality concern:
1. Although the newly added density figures S6 and S7 illustrate the detailed features described in the paper, the quality of Fig. S5B is insufficient to resolve the 28S rRNA cleavage site. Specifically, there appears to be density unaccounted for by the model as shown. I suggest that a more detailed close-up view be shown in place of (or in addition to) one of the panels in Fig. 3, all of which currently illustrate an overall view with the cleavage site hard to distinguish.
2. The statement, "The four resolved states represent a snapshot of the translation elongation cycle," is somewhat misleading, as the four structures represent only a small fraction of an elongation cycle snapshot. The authors could instead state that the structures represent conformationally different states of the elongation cycle. Furthermore, it would be informative to add references (e.g., to review articles) supporting the statement, "These global conformational changes represent the canonical transitions between classical and rotated states observed during eukaryotic elongation."
3. Since the elongation mechanism involving subunit rotation is not limited to eukaryotes, the following statement could be expanded to all organisms in this sentence: "The structural features observed here align with conformational changes observed in the elongation mechanism in eukaryotes."
4. Since the term "universally conserved" refers to all three kingdoms of life, either the word "universally" or "mammalian" should be removed from, "To identify possible species-specific features from the universally conserved architecture of the mammalian ribosomes."
5. In Figure S3, spell out "FSC."
6. Structural models (provided in CIF files) still need attention: (a) base-pairing geometry is disrupted in many peripheral regions (for example, see representative residue 1026, which is nearly perpendicular to the base-pairing plane)—it is particularly important to apply base-pairing restraints in regions with low-resolution density; (b) double-check details of chemical modifications (e.g., m7G1175 shown in Fig. S6 as an m7G, instead features the methyl group attached to C8).

Andrei Korostelev

Reviewer #2

(Remarks to the Author)
The authors have appropriately replied to my previous comments, indicating difficulties in generating the requested data that would go beyond the scope of this study. Moreover, they have now added the two new structures to strengthen their interpretations and conclusions. Therefore, I recommend publication of the revised manuscript.

Reviewer #3

(Remarks to the Author)
I co-reviewed this manuscript with one of the reviewers who provided the listed reports. This is part of the Nature Communications initiative to facilitate training in peer review and to provide appropriate recognition for Early Career Researchers who co-review manuscripts.

Open Access This Peer Review File is licensed under a Creative Commons Attribution 4.0 International License, which permits use, sharing, adaptation, distribution and reproduction in any medium or format, as long as you give appropriate credit to the original author(s) and the source, provide a link to the Creative Commons license, and indicate if changes were made.
In cases where reviewers are anonymous, credit should be given to 'Anonymous Referee' and the source.
The images or other third party material in this Peer Review File are included in the article's Creative Commons license, unless indicated otherwise in a credit line to the material. If material is not included in the article's Creative Commons license and your intended use is not permitted by statutory regulation or exceeds the permitted use, you will need to obtain permission directly from the copyright holder.
To view a copy of this license, visit https://creativecommons.org/licenses/by/4.0/

Point-by-point response to the reviews

Reviewer #1 (Remarks to the Author):

The manuscript by Gul and colleagues reports two cryo-EM structures of the ribosome purified from naked mole rat (NMR) liver. The rationale for this work is that while NMR is a popular animal model in the aging, cancer and other research fields, ribosome structures from this organism have not been reported. Furthermore, the large rRNA is fragmented; this unusual feature, in comparison to other mammals, may result in a peculiarity in the ribosome structure with functional implications. The authors describe two elongation states at 2.7 to 2.9 A average resolution, bound with two tRNAs within a rotated or non-rotated ribosome state. The structures are similar to mouse and human ribosomes, as expected from high sequence conservation. Despite being fragmented, the 28S rRNA and the proteins surrounding the cleavage site are structured similarly to those in non-fragmented homologs, in keeping with high structure conservation. The authors also describe several rRNA modified nucleotides and assign Mg+2 ions coordinated by rRNA. The quality of the cryo-EM data and maps appears high, although additional illustrations are needed to support main findings (see below). As the first report of NMR ribosomes, this study will be a valuable resource.

Thank you very much for their positive assessment of our manuscript. We worked on the improved presentation of our results and added two more structures and multiple display items. Furthermore, we split Figure 2 into Figure 2 and a new Table 1. More detailed response to your review is below.

1. It is somewhat surprising that particle classification resulted in two clean classes. In most studies, multiple ribosome states commonly arise, with different tRNA and translation factor occupancies. The two classes may therefore have sub-stoichiometric tRNAs or mixtures of other states with low-occupancy factors (i.e., poorly visible at high contour levels). Have the authors tried classifications into multiple classes or using masks in different locations, such as the GTPase center? The latter may reveal minor classes with translation factors.

We thank the reviewer for this suggestion. Following this advice, we performed further 3D classification on our dataset. We identified two additional subpopulations: a non-rotated classical pre-translocation state with tRNAs in the A/A, P/P, and E/E sites and a rotated-1 pre-translocation state with tRNAs in the A/A and P/E sites.

These maps had the resulting resolutions of 2.9 and 3.0 Å, respectively. We have also built the atomic models for these maps and deposited them to EMDB/PDB. We added

descriptions of the states in the results section (lines 82-120). And updated the corresponding figures and tables.

2. In the classification figure (S2), add the position of the mask (e.g., in the starting reconstruction).

Thank you, we added the visualization of the mask in the figure, which has now become Figure S1.

3. The key features emphasized in the manuscript are not illustrated as close-up density views with fitted structures. Support these conclusions by corresponding close-up views:
a. the rRNA cleavage site
b. rRNA modifications
c. Mg2+ coordination sites

Thank you, we added Supplementary Figures S5, S6, and S7 showing close-up views of the rRNA cleavage site, representative rRNA modifications, and Mg2+ coordination sites.

4. How the ion densities were unambiguously annotated as Mg2+ (as opposed to other ions)? Describe in Methods and show a few examples of densities with coordination geometry, as noted above.

Thank you for the question, we clarified it in the methods section. The identification of the Mg2+ ions was based on three criteria: (1) strong spherical density, (2) appropriate distances to phosphate oxygens (~2.0–2.2 Å), and (3) structural conservation with high-resolution mouse and human ribosome structures (methods section, lines 380-383). We also provided examples of coordination geometry in the new figures mentioned above (Supplementary Figure S7).

5. A mouse ribosome structure was used as a starting model; explain in Methods how the NMR sequences for rRNA and proteins were traced, and which sequences were used for reference.

We generated the specific NMR sequences using the mHetGlaV2, pHetGlaV2, mHetGlaV3 and pHetGlaV3 genome assemblies. The homology models were initially created by threading the NMR sequences onto the mouse backbone (PDB: 7CPU), followed by manual adjustment in Coot to fit the specific NMR density. We added these details to the Methods section (lines 365-368).

6. Due to compositional heterogeneity, which tRNA species were chosen to fit best into densities? Explain in Methods, and provide model-density close-up views.

Due to the compositional heterogeneity of tRNAs in our endogenous lysate-purified ribosomes, the density at the A, P, and E sites represents an average of multiple tRNA species. To model this, we utilized the high-resolution tRNA coordinates from a previously published mouse ribosome structure (PDB: 7CPU), as the mouse and NMR tRNA backbones are highly conserved. We utilized this model as a representative to fit the backbone density, which is well-resolved. We did not attempt to model specific side chains for the variable regions of the tRNA. We have clarified this in the Methods (lines 374-380) and added a close-up of the tRNA density fitting in Supplementary Figure S5.

7. In the data/model statistics table, report the CCs between structural models and maps.
Andrei Korostelev

Thank you, we reported the CC values in Supplementary Table S1 for all our structures.

Reviewer #2 (Remarks to the Author):

The manuscript by Gul and coworders reports two cryo-EM structures of the naked mole-rat (NMR, Heterocephalus glaber) cytosolic ribosome captured in two elongation states: a pre-translocation state (80S–A/P- and P/E-tRNAs) and a post-translocation state (80S–P- and E-tRNAs). The NMR is of particular interest due to its exceptional lifespan, resistance to age-related diseases, and tolerance to various stress conditions. Previous work (Azpurua et al., 2013) demonstrated that the 28S rRNA in NMR is cleaved into two fragments via two cuts within the D6 expansion segment, excising a ~260 nt fragment. That study also showed that although NMR fibroblasts exhibit translation rates comparable to mouse fibroblasts, their translation fidelity is significantly higher, yet this increase could not be directly attributed to the unique 28S rRNA cleavage. Nonetheless, both the unusual rRNA processing and elevated translational fidelity are attractive underlying features that offer unique biology to this organism. The current manuscript aims to characterize the structural integrity of NMR ribosomes harboring the cleaved 28S rRNA and to address how this feature might influence translation fidelity. The authors' main observations and conclusions can be summarized as follows:
1. The conformational changes observed between the two cryo-EM structures are consistent with the canonical elongation cycle described for other eukaryotic ribosomes.
2. The overall architecture of the NMR ribosome is highly similar to that of mouse and human cytosolic ribosomes.

3. Despite the cleavage in the 28S rRNA, the local environment surrounding the break, including the positions of neighboring ribosomal proteins uL4, eL6, eL18, eL28, and uL30, is essentially indistinguishable from that in the mouse ribosome with intact 28S rRNA. Thus, the 28S rRNA cleavage does not appear to induce detectable structural rearrangements in its immediate neighborhood.

Overall, the study provides a structural “snapshot” of the previously described 28S rRNA break within the context of intact 80S ribosomes, thereby confirming that the fragmentation is compatible with a structurally canonical translational apparatus. However, beyond visualizing this rRNA cleavage in situ, the work has limited impact on our mechanistic understanding of how this feature might influence translation fidelity, stress resistance, or other unique physiological traits of the NMR. Further structural analyses of the NMR ribosomes bound to cognate and near-cognate tRNAs, analogous to the work of Loveland et al., (2017), could directly test whether rRNA fragmentation contributes to the reported increase in translational fidelity.

In its current form, the manuscript essentially confirms that (i) the NMR ribosomes are structurally very similar to those of mouse and human, and (ii) the 28S rRNA break does not cause any obvious local structural perturbations. While these are useful descriptive observations, they provide only a modest advance beyond the earlier biochemical characterization and do not yet substantively address the biological significance of the 28S rRNA fragmentation.

We thank the reviewer for their remarks and their insightful suggestion. We agree that the study by Loveland et al. (2017) from the group of reviewer 1 provides an elegant structural basis for translation fidelity in bacterial ribosomes, and that defining the structural mechanism of fidelity in the naked mole-rat would be of high interest.

However, performing analogous structural analyses on NMR ribosomes is not feasible for several reasons. First, the Loveland et al. study utilized an *in vitro* reconstituted system comprising purified *E. coli* 70S subunits, overexpressed and purified EF-Tu, and specific aminoacylated tRNAs trapped with the non-hydrolyzable GTP analog GDPCP. Bacterial reconstitution systems are well-established and robust. In contrast, *in vitro* reconstitution of **mammalian** translation initiation and decoding for high-resolution cryo-EM requires a complex assembly of eukaryotic initiation and elongation factors. Establishing such a reconstituted system for a non-canonical organism such as the naked mole-rat is a major undertaking that extends far beyond the current descriptive characterization.

The focus of our study, however, is the structural integrity of endogenous NMR ribosomes purified directly from tissues. Unlike the controlled *in vitro* setup used by Loveland and colleagues, which allows for the precise saturation of the A site with specific cognate or

near-cognate tRNAs, our endogenous samples contain a heterogeneous mixture of mRNA and tRNAs. Trapping specific decoding intermediates requires precise biochemical control that is currently only feasible in highly optimized model systems, like *E. coli* or yeast (PMID: 26270393, 38247969), and has not yet been established for more complex systems like NMR.

The result of our manuscript is the first structural evidence that the unique 28S rRNA cleavage in NMR ribosomes does not compromise the overall structural integrity of the 80S subunit during the canonical elongation cycle. We believe our current structures clearly demonstrate this by showing that the cleavage site remains stable and indistinguishable from the uncleaved mouse ribosome.

We agree that determining the structure of NMR decoding intermediates is an exciting future direction to understand their longevity-associated fidelity; we believe it represents a technically demanding project separate from the structural characterization presented here.

We further extended our analysis by determining two more structures of NMR ribosomes in the canonical states of the elongation cycle.

Reviewer #3 (Remarks to the Author):

I co-reviewed this manuscript with one of the reviewers who provided the listed reports. This is part of the Nature Communications initiative to facilitate training in peer review and to provide appropriate recognition for Early Career Researchers who co-review manuscripts.

We thank the reviewer for the evaluation of our manuscript.

RESPONSE TO REVIEWERS

Our response is in blue

Reviewer #1 (Remarks to the Author):

In this revised manuscript, additional analyses and updated text and figures address most of the concerns I had raised. The following suggestions address the remaining minor manuscript concerns and a structure quality concern:

Thank you very much for your detailed evaluation of our manuscript and the structures.

1. Although the newly added density figures S6 and S7 illustrate the detailed features described in the paper, the quality of Fig. S5B is insufficient to resolve the 28S rRNA cleavage site. Specifically, there appears to be density unaccounted for by the model as shown. I suggest that a more detailed close-up view be shown in place of (or in addition to) one of the panels in Fig. 3, all of which currently illustrate an overall view with the cleavage site hard to distinguish.

Thank you for this suggestion. We updated Fig. S5B with a detailed close-up view of the cleavage site and marked the flanking fragments with color.

The unaccounted density corresponds to a small rRNA fragment in the D6 region whose nucleotide sequence is not present in the available NMR genome assemblies. Therefore, we built four adenine nucleotides in all four models to have a more complete atomic model around the cleavage site.

2. The statement, “The four resolved states represent a snapshot of the translation elongation cycle,” is somewhat misleading, as the four structures represent only a small fraction of an elongation cycle snapshot. The authors could instead state that the structures represent conformationally different states of the elongation cycle. Furthermore, it would be informative to add references (e.g., to review articles) supporting the statement, “These global conformational changes represent the canonical transitions between classical and rotated states observed during eukaryotic elongation.”

Thank you, we agree and changed the statement accordingly (lines 97-98). It reads:

“The four resolved states represent conformationally distinct states of the translation elongation cycle in the naked mole-rat (Figure 1A).”

References were added for the second statement.

Furthermore, we made a series of minor modifications throughout the text to make our statements more precise.

3. Since the elongation mechanism involving subunit rotation is not limited to eukaryotes, the following statement could be expanded to all organisms in this sentence: "The structural features observed here align with conformational changes observed in the elongation mechanism in eukaryotes."

Thank you, we updated the statement (lines 121-122), it reads: "The structural features observed here align with conformational changes observed in the translation elongation mechanism."

4. Since the term "universally conserved" refers to all three kingdoms of life, either the word "universally" or "mammalian" should be removed from, "To identify possible species-specific features from the universally conserved architecture of the mammalian ribosomes."

Thank you, we removed the word "universally" from the sentence (line 137).

5. In Figure S3, spell out "FSC."

Thank you, we added the axis names, and FSC was spelled out in the figure legend.

6. Structural models (provided in CIF files) still need attention: (a) base-pairing geometry is disrupted in many peripheral regions (for example, see representative residue 1026, which is nearly perpendicular to the base-pairing plane)—it is particularly important to apply base-pairing restraints in regions with low-resolution density; (b) double-check details of chemical modifications (e.g., m7G1175 shown in Fig. S6 as an m7G, instead features the methyl group attached to C8).

Thank you for the detailed examination of our maps and models.

We addressed this in two steps. First, we manually inspected and corrected the most severely misfit residues in Coot, including residue 1026, which showed improved base orientation after manual rebuilding in all affected states.

Second, we re-refined all four models with RNA secondary structure restraints explicitly enabled in Phenix *real_space_refine*, including base-pair hydrogen bond restraints. Sugar pucker outliers were reduced in the models.

We double-checked the chemical modifications. m7G1175 represents the N7-methylguanosine modification, and the corresponding figure panel indicates the methyl group attached to N7.

We re-uploaded our models to the PDB database and to the submission.

Andrei Korostelev

Reviewer #2 (Remarks to the Author):

The authors have appropriately replied to my previous comments, indicating difficulties in generating the requested data that would go beyond the scope of this study. Moreover, they have now added the two new structures to strengthen their interpretations and conclusions. Therefore, I recommend publication of the revised manuscript.

We thank the reviewer for the constructive comments, positive assessment, and recommendation for publication.

Reviewer #3 (Remarks to the Author):

I co-reviewed this manuscript with one of the reviewers who provided the listed reports. This is part of the Nature Communications initiative to facilitate training in peer review and to provide appropriate recognition for Early Career Researchers who co-review manuscripts

We thank the reviewer for the contribution to the review process that helped us to improve the manuscript.
